# Gender Differences of Dementia in Response to Intensive Self-Administered Transcranial and Intraocular Near-Infrared Stimulation

**DOI:** 10.7759/cureus.16188

**Published:** 2021-07-05

**Authors:** Xiaoming Qi, Damir Nizamutdinov, Marvin H Berman, Gordon Dougal, Paul L Chazot, Erxi Wu, Alan B Stevens, S. Stephen Yi, Jason H Huang

**Affiliations:** 1 Neurosurgery, Baylor Scott & White Health, Temple, USA; 2 Neuropsychology, QuietMIND Foundation, Elkins Park, USA; 3 Chief Executive Officer, Maculume Limited, Spennymoor, GBR; 4 Biomedical Sciences, Durham University, Durham, GBR; 5 Gerontology, Baylor Scott & White Health Research Institute, Temple, USA; 6 Oncology, The University of Texas at Austin, Dell Medical School, Austin, USA; 7 Neurosurgery, Baylor Scott & White Medical Center, Temple, USA

**Keywords:** transcranial near-infrared, photobiomodulation, sex, dementia, alzheimer's disease, alzheimer related disease

## Abstract

Background

Transcranial near-infrared (tNIR) stimulation was proven to be a safe, reliable, and effective treatment for cognitive and behavioral symptoms of dementia. Dementia patients of different genders differ in terms of gross anatomy, biochemistry, genetic profile, clinical presentations, and socio-psychological status. Studies of the tNIR effect on dementia have thus far been gender-neutral, with dementia subjects being grouped based on diagnoses or dementia severity. This trial hereby investigated how dementia subjects of different sex respond to tNIR treatment.

Methods

A total of 60 patient-caregiver dyads were enrolled and randomized to this double-blind, sham-controlled clinical trial. The tNIR light has a wavelength of 1,060 nm to 1,080 nm and was delivered via a photobiomodulation (PBM) unit. The active PBM unit emits near-infrared (NIR) light while the sham unit does not. The treatment consists of a six-minute tNIR light stimulation session twice daily for eight weeks. Neuropsychological assessments conducted at baseline (week 0) and endline (week 8) were compared within the female and male group and between different sex, respectively.

Results

Over the course of treatment, active-arm female subjects had a 20.2% improvement in Mini‐Mental State Exam (MMSE) (mean 4.8 points increase, p < 0.001) and active-arm male cohort had 19.3% improvement (p < 0.001). Control-arm female subjects had a 6.5% improvement in MMSE (mean 1.5 points increase, p < 0.03) and control-arm male subjects had 5.9% improvement (p = 0.35) with no significant differences in the mean MMSE between female and male subjects in both arms respectively. Other comparison of assessments including Clock Copying and Drawing Test, Logical Memory Test - immediate and delayed recall yielded nominal but not statistically significant differences. No significant differences were observed in the mean MMSE between female and male subjects in both arms respectively before treatment implementation (active arm, p = 0.12; control arm, p = 0.50) at week 0, or after treatment completion (active arm, p = 0.11; control arm, p = 0.74) at week 8.

Conclusion

Despite differences between female and male dementia subjects, the response to tNIR light stimulation does not demonstrate gender-based differences. Further studies are warranted to refine the tNIR treatment protocol for subjects suffering from dementia or dementia-related symptoms.

## Introduction

Alzheimer’s disease (AD) and other forms of dementia have, until recently, been thought to be an irreversible, progressive syndrome characterized by neurocognitive impairment. Ten percent of the U.S. population age 65 and above has been diagnosed with AD - the most common cause of dementia [1]. Apart from AD, dementia can also be caused by Alzheimer’s disease-related dementias (ADRD). This includes mild cognitive impairment (MCI) and less frequent vascular dementia (VaD), Lewy body dementia (LBD), frontotemporal dementia (FTD), and Parkinson-related dementia (PRD). The number of people with AD or ADRD is expected to increase and escalate dramatically in the coming years with aging of the baby boomer generation [1]. Clinically, people with dementia present with a cluster of neuropsychiatric and neuropsychological signs and symptoms. With impairments of memory, executive function, and emotional and behavioral stability, dementia significantly compromises the quality of life and the capacity to function independently, thus increasing the burden on family, caregivers and the community.

Photobiomodulation (PBM) therapy employing transcranial near-infrared (tNIR) stimulation has recently been shown to be a safe, low-cost, easily deployed and reliable treatment for both cognitive and behavioral symptoms of dementia. The tNIR at 1072 nm proves to lower brain beta-amyloid protein in AD mouse model, and improves memory performance and emotional response [2,3]. Photic stimulation of cortical mitochondria is now widely accepted as a primary mechanism of action, i.e., elevating intracellular adenosine triphosphate (ATP) by stimulating cytochrome c oxidase (CCO) - a chromophore that reacts to light within the 700 nm-1,100 nm spectrum. The increased ATP is of direct benefit to people with dementia, as many neuropathologies are characterized by markedly decreased ATP [4-6]. tNIR stimulation also improves endothelial flexibility thus improving cortical perfusion and regional cerebral blood flow, thereby increasing oxygenation of cortical white matter by upregulating the production of nitric oxide (NO), a potent vasodilator [7-9]. Additionally, tNIR stimulation mitigates inflammatory reaction by modulating the NF-κB system [10], tumor necrosis factor (TNFα), and other inflammatory cytokines in brain parenchyma [11]; activates anti-apoptotic, and anti-senescence cascades via complex regulation of transcription factors [12-15]; and promotes neurogenesis, synaptogenesis and neurogenesis through activation of brain-derived neurotrophic factor (BDNF) [16,17]. Numerous clinical trials utilizing tNIR stimulation for the treatment of neurological and neuropsychiatric disorders have been proven safe and effective [18-23].

The brains of male and female differ anatomically in volume, cerebral blood flow, and proportions of white and grey matter [24]. Neurochemically, there are gender differences in the level of dopaminergic, serotonergic, cholinergic and GABAergic markers [25,26]. Hormonally, female has higher level of neuroprotective estrogen and progesterone compared to male until menopause, when the hormone levels become relatively equivalent. Genetically, apolipoprotein E4 (APOE4) allele has a worse impact on women than on men in sporadic Alzheimer’s Disease [27,28]. Clinically, neurocognitive and other neuropsychiatric manifestations change dependent on patient gender.

Studies of PBM’s effect on dementia have thus far been sex-neutral, with dementia subjects being grouped primarily based on diagnoses or disease severity. With the question of how gender may influence the response to tNIR therapy, this study examined the response of female and male subjects diagnosed with early- to mid-stage dementia to intensive tNIR stimulation.

## Materials and methods

This study was a double-blind, randomized and sham-controlled trial, conducted at Baylor Scott & White Health (BSWH) Medical Center in Temple, TX. The study protocol was approved by and conducted under the supervision of the Baylor Scott & White Research Institute Institutional Review Boards. The study was funded by Clarke Brain Institute, a non-profit charitable foundation.

The study population consists of persons independently diagnosed with mild to moderate AD/ADRD, and their primary caregivers. Inclusion criteria: (1) males and females, age 50-85; (2) persons living with mild to moderate AD/ADRD (MMSE = 15-24); (3) good general health status other than dementia. Exclusion criteria: (1) history of actively growing or recurrent intracranial neoplasms; (2) history of epilepsy; (3) history of acute ischemic stroke. ADRD was defined as cognitive deficits caused by MCI, LBD, FTD, Parkinson-associated dementia (PD), VaD, and non-specific dementia (NSD).

Sixty patient-caregiver dyads were enrolled and randomized to the active arm (active treatment) or control arm (sham treatment) at a 2:1 ratio. Subjects, caregivers, and assessors were masked as to randomization. Subjects received either an active or a sham portable PBM unit to be used at home twice daily. Both active and sham PBM units have 12 LED modules covering the skull and two retractable modules to provide intraocular stimulation. Each cranial module contains 70 LEDs and each eye module contains 14 LEDs. The sham PBM units were identical in design, except that it does not emit NIR light. Subjects and investigators could not tell which device was active or placebo because infrared light at 1068nm is invisible to the naked eye. The active PBM unit emitted NIR light with a wavelength of 1,060-1,080 nm, and 15,000 mW, irradiance or power density is 23.1 mW/cm^2^, ~650 cm^2^ per treatment area. The treatment protocol consisted of twice-daily six-minute stimulation sessions conducted at home over eight consecutive weeks.

Two neuropsychological assessments were conducted eight weeks apart. The baseline assessment at week 0 was performed on the first day of the treatment, the endline assessment at week 8 was performed on the last day of the treatment session. The following were used for cognitive functioning assessment: (1) Mini‐Mental State Exam (MMSE): the MMSE is a global cognitive function measurement. It is a 30-point scale assessing orientation, memory, attention, concentration, language and visuospatial function. (2) Clock Drawing Test (CDT): in the CDT, patients were verbally instructed to draw an analog clock reflecting a specific time. (3) Clock Copying Test (CPT): patients were given an analog clock image and instructed to draw a copy. (4) Logical Memory Test- immediate recall (LMT-I): Subjects would hear a story, and were instructed to recall the story immediately. (5) Logical Memory Test - delayed recall (LMT-II): patients were instructed to recall the story again 30 minutes but no more than 40 minutes after LMT-I. (6) Trail Making Test A & B: in the TMT part A, subjects were instructed to connect randomly positioned circled numbers in their numerical order from 1 to 25. In the TMT Part B, a piece of paper with both circled numbers (1-12) and letters (A-L) was provided to the subject. The subject would connect the circled numbers and letters in order like this: 1-A-2-B until reaching 12-L-13. (7) Boston Naming Test (BNT): subjects were shown 30 different items, and were instructed to name each shown item. (8) WAIS-R Digit Symbol Substitution Test: a daily treatment diary was kept by the corresponding caregivers. All evaluations were completed by the same assessor across the study.

Analysis of variance (one-way ANOVA) was used for evaluation of mean differences between week 0 and week 8 assessment outcomes. Schumann method was used to score Clock Drawing Test and Clock Copying Test. Student t-test was used to assess the mean differences of female and male subjects at week 0 and week 8 within the same study arm. A p-value less than 0.05 (p < 0.05) was deemed as statistically significant.

## Results

Sixty dyads of persons living with AD/ADRD and their caregivers were enrolled and randomized. Three subjects in the control arm and one subject in the active arm withdrew from the trial for reasons unrelated to tNIR light therapy. Fifty-six subjects had completed the eight-week treatment protocol. The general demographics are shown in Table 1.

**Table 1:** Demographics of subjects completed the eight-week treatment protocol.

	Number	Age (years)	SD	Percentage	Sham treatment	Active treatment
Male	32	74 (54-86)	7.5	57.10%	9	23
Female	24	73.8 (51-85)	8.4	42.90%	8	16
Total	56				17	39

A total of 39 subjects were randomized to the active arm, and 17 were randomized to the control arm. No adverse effects were reported by subjects or caregivers that were associated with tNIR treatment during or after completion of the study.

Generally, in response to the tNIR stimulation, subjects in the active arm reported having more energy, elevated mood and less anxiety, and better physical and mental involvement in daily activities. These responses began after two to three weeks and only had been observed among those receiving active treatment.

The caregiver cohort (N = 60) consisted of 44 females (73.3%) and 16 males (26.7%). Of the total 24 female subjects, 33.3% (n = 8) were cared for by a close female family member; and 66.7% (n = 16) were cared for by a close male family member. All enrolled male subjects (n = 36) were cared for by a close female family member.

The results of neuropsychological assessments at week 0 (baseline) and week 8 (end of treatment) were compared between the active arm and control arm within female and male groups. The findings were noted as follows.

*Mini-Mental State Exam (MMSE).* In the active arm of the female cohort, the average MMSE score increased from 23.6 ± 1.9 at week 0 to 28.3 ± 1.6 at week 8 (p < 0.001), which was a 4.8 points improvement (20.2% increase) over the course of treatment. In the control arm of the female cohort, the average MMSE score changed from 23.0 ± 0.9 at week 0 to 24.5 ± 1.5 at week 8 (p = 0.03), which was a 1.5 points improvement (6.5% increase) over the course of treatment (Table 2).

**Table 2:** Mini-Mental State Exam, Clock Drawing, Clock Copying Tests, and Logical Memory Test I and II in females. MMSE: Mini-Mental State Exam; CDT: Clock Drawing Test; CCT: Clock Copying Test; LMT-I: Logical Memory Test – Immediate total story unit recall; LMT-II: Logical Memory Test – Delayed total story unit recall; SD: standard deviation. *p-value < 0.05; ***p-value < 0.001.

Subtest	Female sham treatment					Female active treatment				
	Before		After			Before		After		
	Mean	SD	Mean	SD	p-value	Mean	SD	Mean	SD	p-value
MMSE	23.0	0.9	24.5	1.5	0.0313*	23.6	1.9	28.3	1.6	1.7E-08***
CDT	4.3	1.4	3.9	1.5	0.6066	3.8	1.3	4.3	1.0	0.3522
CCT	5.0	0	5.0	0	0.3343	4.7	1.0	4.8	0.8	0.8460
LMT-I	5.5	5.5	4.3	4.9	0.6376	8.1	6.8	10.4	6.5	0.3453
LMT-II	1.5	2.9	1.3	3.5	0.8806	5.9	6.5	7.9	7.5	0.4384

In the active arm of the male cohort, the average MMSE score improved from 22.4 ± 2.6 at week 0 to 26.7 ± 3.7 (p < 0.001) at week 8, which was a 4.3 points improvement (19.3% increase) over the course of the treatment. In the control arm male cohort, the average MMSE score changed from 23.4 ± 2.2 at week 0 to 24.8 ± 3.4 (p = 0.35) at week 8, which was a 1.4 points improvement (5.9% increase) over the course of treatment (Table 3).

**Table 3:** Mini-Mental State Exam, Clock Drawing, Clock Copying Tests, and Logical Memory Test I and II in males. MMSE: Mini-Mental State Exam; CDT: Clock Drawing Test; CCT: Clock Copying Test; LMT-I: Logical Memory Test – Immediate total story unit recall; LMT-II: Logical Memory Test – Delayed total story unit recall; SD: standard deviation. ***p-value < 0.001.

Subtest	Male sham treatment					Male active treatment				
	Before		After			Before		After		
	Mean	SD	Mean	SD	p-value	Mean	SD	Mean	SD	p-value
MMSE	23.4	2.2	24.8	3.4	0.3503	22.4	2.6	26.7	3.7	5.7E-05***
CDT	3.4	1.4	3.9	1.5	0.4967	3.4	1.4	3.8	1.4	0.2878
CCT	4.5	1.1	4.4	1.4	0.8444	4.1	1.2	4.5	1.1	0.2353
LMT-I	9.4	3.8	7.6	3.7	0.3675	8.9	5.4	12.3	7.5	0.0925
LMT-II	7.1	4.2	4.1	4.2	0.1741	6.9	5.5	10.7	8.1	0.0773

No significant differences were observed in the mean MMSE between female and male subjects in both arms respectively before treatment implementation (active arm, p = 0.12; control arm, p = 0.50) at week 0, or after treatment completion (active arm, p = 0.11; control arm, p = 0.74) at week 8 (Table 4).

**Table 4:** Mini-Mental State Exam, Clock Drawing, Clock Copying Tests, and Logical Memory Test I and II in the active arm in both sexes. MMSE: Mini-Mental State Exam; CDT: Clock Drawing Test; CCT: Clock Copying Test; LMT-I: Logical Memory Test – Immediate total story unit recall; LMT-II: Logical Memory Test – Delayed total story unit recall; SD: standard deviation.

Subtests	Active treatment week 0					Active treatment week 8				
	Female		Male			Female		Male		
	Mean	SD	Mean	SD	p-value	Mean	SD	Mean	SD	p-value
MMSE	23.6	1.9	22.4	2.6	0.1240	28.3	1.6	26.7	3.7	0.1132
CDT	3.8	1.3	3.4	1.4	0.3722	4.3	1	3.8	1.4	0.2281
CCT	4.7	1.0	4.1	1.2	0.1093	4.8	0.8	4.5	1.1	0.3577
LMT-I	8.1	6.8	8.9	5.4	0.6848	10.4	6.5	12.3	7.5	0.4171
LMT-II	5.9	6.5	6.9	5.5	0.6073	7.9	7.5	10.7	8.1	0.2810

Baseline mean MMSE between active and control arm were compared within female and male groups, and no statistical significant differences were observed (Table 5).

**Table 5:** Mini-Mental State Exam, Clock Drawing and Copying Tests, and Logical Memory Test I and II at baseline in both sexes. MMSE: Mini-Mental State Exam; CDT: Clock Drawing Test; CCT: Clock Copying Test; LMT-I: Logical Memory Test – Immediate total story unit recall; LMT-II: Logical Memory Test – Delayed total story unit recall; SD: standard deviation.

Subtests	Female week 0					Male week 0				
	Sham		Active			Sham		Active		
	Mean	SD	Mean	SD	p-value	Mean	SD	Mean	SD	p-value
MMSE	23.0	0.9	23.6	1.9	0.4098	23.4	2.2	22.4	2.6	0.3170
CDT	4.0	1.4	3.8	1.3	0.7322	3.4	1.4	3.4	1.4	1.000
CCT	5.0	0	4.7	1.0	0.4105	4.5	1.1	4.1	1.2	0.3931
LMT-I	5.5	5.5	8.1	6.8	0.3594	9.4	3.8	8.9	5.4	0.8019
LMT-II	1.5	2.9	5.9	6.5	0.0838	7.1	4.2	6.9	5.5	0.9225

*Clock Drawing Test (CDT)*. Positive trends of improvement in CDT scores were observed. For female subjects in the active arm, the mean CDT score went up from 3.8 ± 1.3 at week 0 to 4.3 ± 1.0 (p = 0.35) at week 8. This was a 0.4 points improvement (11.5% increase) over the course of treatment. In the female control arm group, patients demonstrated a trend of decrease in CDT, with an average shift from 4.3 ± 1.4 down to 3.9 ± 1.5 (p = 0.61), which was a 0.4 score points and 8.8% decrease in mean CDT score throughout sham treatment (Table 2). Male subjects in the active arm had a positive trend of improvement in CDT scores, with the mean CDT value going up from 3.4 ± 1.4 at week 0 to 3.8 ± 1.4 (p = .29) at week 8. This was a 0.5 points improvement (13.5% increase) over the course of treatment. Male subjects in the control arm demonstrated a trend of decrease in CDT scores, with an average shift from 3.4 ± 1.4 to 3.9 ± 1.5 (p = 0.50), which was 0.5 score points (14.8% increase) in mean CDT score throughout sham treatment (Table 3). No significant differences were observed in the CDT score between female and male subjects in the active arm before treatment implementation (p= 0.37) at week 0 or after treatment completion (p = 0.23) at week 8 (Table 4). Baseline CDT scores between active and control arm were compared within female and male groups, and no statistical significant differences were observed (Table 5).

*Clock Copying Test (CCT)*. Female subjects in the active arm showed very little difference, with a mean CCT score changing from 4.7 ± 1.0 at week 0 to 4.8 ± 0.8 (p = 0.85) at week 8, which was a 1.3% increase over the course of treatment. Female subjects in the control arm showed no change in CCT score; both CCT mean scores were 5.0 ± 0.0 (0% change, Table 2). Male subjects in the active arm showed a positive trend of CCT. The average score went up from 4.1 ± 1.2 at week 0 to 4.5 ± 1.1 (p = 0.24) at week 8. This was a 10.0% increase within the time of treatment. Male subjects in the control arm, however, demonstrated a downward trend of CCT score. The average score decreased from 4.5 ± 1.1 to 4.4 ± 1.4 (p = 0.84) over the course of treatment, which was a 2.8% decline (Table 3). No significant differences were observed in the CCT score between female and male subjects in the active arm before treatment implementation (p = 0.11) at week 0 or after treatment completion (p = 0.36) at week 8 (Table 4). Baseline CCT scores between active and control arm were compared within female and male groups, and no statistical significant differences were observed (Table 5).

*Logical Memory Test- immediate recall (LMT-I)*. Female active arm subjects demonstrated improved performance in total story passage recall. There was a 2.3 points (27.7%) increment over the course of treatment, increasing from 8.1 ± 6.8 at week 0 to 10.4 ± 6.5 (p = 0.35) at week 8. In comparison, female control arm subjects demonstrated a decrease of 1.3 points (22.7%) decrease over the course of treatment in the performance for story recall, declining from 5.5 ± 5.5 at week 0 to 4.3 ± 4.9 (p = 0.64) at week 8 (Table 2). Male active arm subjects increased by 3.4 score points (38.5%) from 8.9 ± 5.4 at week 0 to 12.3 ± 7.5 (p = 0.10) at week 8. Male control arm subjects' performance declined by 1.8 score points (18.7%) from 9.4 ± 3.8 down to 7.6 ± 3.7 (p = 0.37) over eight weeks sham treatment (Table 3). No significant differences were observed in the LMT-I score between female and male subjects in the active arm before treatment implementation (p = 0.68) at week 0 or after treatment completion (p = 0.42) at week 8 (Table 4). Baseline LMT-I scores between active and control arm were compared within female and male groups, and no statistical significant differences were observed (Table 5).

*Logical Memory Test - delayed recall (LMT-II)*. Active device-treated female subjects also demonstrated improved performance by an increase of 1.9 points (32.6%) from 5.9 ± 6.5 at week 0 to 7.9 ± 7.5 (p = 0.44) at week 8. Sham-treated female subjects demonstrated a decrease in performance similar to the LMT-I test with a 0.25 points (16.7%) decline from 1.5 ± 2.9 to 1.3 ± 3.5 (p = 0.88) over the course of sham treatment (Table 2).

Delayed total story passage recall in male subjects treated with active tNIR light demonstrated even greater performance improvement than the female subjects. The average scores of LMT-II increased from 6.9 ± 5.5 at week 0 to 10.7 ± 8.1 (p = 0.08) at week 8, which was 3.8 points (54.3%) increase over the treatment course. As opposed to the male control arm, the average scores decreased from 7.1 ± 4.2 to 4.1 ± 4.2 (p = 0.17), which was a 3.0 points (42.1%) decline within the treatment course (Table 3). No significant differences were observed in the LMT-II score between female and male subjects in the active arm before treatment implementation (p = 0.61) at week 0 or after treatment completion (p = 0.28) at week 8 (Table 4). Baseline LMT-II scores between active and control arm were compared within female and male groups, and no statistical significant differences were observed (Table 5).

*Trail Making Test A*. Active-arm female subjects did not demonstrate a significant change in performance time. The average time to completion of a task changed from 48.5 ± 15.9 seconds at week 0 to 48.6 ± 31.7 seconds (p = 0.98) at week 8, which was only a 0.1-second difference. Control-arm female subjects demonstrated 10.3 seconds (16.0%) improvement in time completion of the task, changing from 64.1 ± 37.5 seconds to 53.9 ± 33.0 seconds (p = 0.59) over the course of treatment.

Active-arm male subjects demonstrated decreased average time of completion from 68.2 ± 31.7 seconds at week 0 to 59.9 ± 26.5 seconds (p = 0.40) at week 8, which was 8.3 seconds (12.1%) faster. Control-arm male subjects demonstrated an increase in an overall time of performance for this test by 9.3 seconds (10.7%) on average over the course of sham treatment. The average time of completion in this group changed from 86.6 ± 41.2 seconds to 95.9 ± 49.7 seconds (p = 0.69) at the end of treatment.

*Trail Making Test B*. More comprehensive and demanding in execution than Test A, Test B is given twice as much time to complete. Active-arm female subjects were on average 29.4 seconds faster in the execution of Test B (20.6% of improvement), with the average time for completion decreasing from 143.0 ± 76.9 seconds at week 0 to 113.6 ± 45.5 seconds (p = 0.24) at week 8. Control-arm female subjects were 11.9 seconds slower in completion of Test B (6.5% of decline) on average, shifting from 182.1 ± 89.4 seconds at week 0 to 194.0 ± 92.1 seconds at week 8 (p = 0.81).

Active-arm male subjects demonstrated unsubstantial change for Trail Making Test B. Average completion time changed from 179.3 ± 81.2 seconds to 181.3 ± 71.2 seconds (p = 0.93), which was just a 1.1% difference. Control-arm male subjects also demonstrated an unsubstantial change of 1.5% improvement, changing from 223.1 ± 97.3 seconds at week 0 to 219.9 ± 98.6 seconds at week 8 (p = 0.95).

*Boston Naming Test (BNT)*. In the active arm, female subjects had better performance than male subjects. The average score in active-arm female subjects female improved from 23.2 ± 5.7 at week 0 to 25.8 ± 4.9 (p = 0.18) at week 8, which was a 2.6 points increase (11.1%) increase. The certain individual responded substantially to the treatment with a 28.6% improvement. Control-arm female subjects demonstrated no substantial change in BNT performance from 24.0 ± 6.6 to 24.4 ± 6.1 (p = 0.90), which was only 0.4 points (1.8%) difference.

Active-arm male subjects demonstrated an average score change from 25.6 ± 2.9 points to 27.2 ± 2.9 points (p = 0.07) over the course of treatment, which was a 1.6 score points (6.2%) difference. Control-arm male subjects demonstrated a 1.5 score points (6.4%) change over the treatment course, shifting from 23.4 ± 2.6 at week 0 to 24.9 ± 3.6 (p = 0.35) at week 8.

*WAIS‐R Digit Symbol Substitution Test*. Performance of active-arm female subjects improved from 31.9 ± 14.1 at week 0 to 37.1 ± 16.2 at week 8 (p = 0.34), which was a 5.2 points (16.3%) increase. Control-arm female subjects did not show substantial improvements in performance, changing from 39.1 ± 8.6 at week 0 to 39.4 ± 10.7 (p = 0.95) at week 8, which was a mere 0.3 points (0.7%) difference over the course of sham treatment.

Active-arm male subjects had improved performance increasing from 26.8 ± 10.5 to 29.2 ± 9.9 (p = 0.48) over eight weeks of active treatment. This resulted in 2.4 score points (8.8%) of improvement. However, sham-treated male subjects also displayed similar performance improvement, increasing from 19.8 ± 12.1 to 21.1 ± 13.1 points (p = 0.83), which was 1.4 points (6.9%) of improvement.

## Discussion

In this double-blind, randomized clinical trial, we prospectively compared response to tNIR stimulation in dementia patients of different genders. At baseline (week 0), there were no differences in the performance of MMSE, CDT, CCT, LMT-I, and LMT-II between active arm and control arm in female or male subjects. This indicates that the active arm and control arm subjects did not differ at baseline regardless of gender. At endline (week 8), both female and male active arm subjects performed better than their control arm counterparts in MMSE respectively. The MMSE consists of elements assessing various cognitive realms and correlates with disease progression. MMSE has been widely used in both clinical practice and clinical trials for dementia screening and progression monitoring [29-31]. The statistically significant improvements observed in this study indicate that subjects of both genders can respond positively to the tNIR stimulation. Based on the MMSE scores, there were no statistically significant differences observed between female and male subjects within the active treatment cohort, both at baseline and treatment completion. This suggests that tNIR stimulation does not confer differential impact based on sex despite recognized anatomical, biochemical, and culturally determined gender role differences. The tNIR can enhance mitochondrial function and cellular metabolism [32,33], increase regional cerebral blood flow [8], increase axonal transport [34,35], mitigate inflammatory reaction and oxidative stress [36,37], and promote synaptogenesis [38]. We believe it was through the aforementioned mechanisms of action that tNIR stimulation had a positive disease-modifying impact on dementia-related symptoms in this study.

The brain differs between men and women in terms of structure, function and neurochemistry [24]. Women have higher global cerebral blood flow while men have larger overall brain volume; women have a larger percentage of gray matter compared to men, who have a larger proportion of white matter [39-41]. Differences between male and female have also been observed in dopaminergic, serotonergic, cholinergic and GABAergic markers, indicating distinct neurochemical gender profiles [25,26]. The mosaic inactivation of X-chromosome in females also modifies brain function [42-44]. On a socio-psychological level, gender variations of lifestyles and behaviors may affect brain functionality [45]. These all could influence differences in prevalence, onset and course of dementia-related symptoms [46].

Androgens and estrogens may have an effect on cognition and the development and progression of dementia [47]. Rocca et al. found that pre-menopause women who have undergone bilateral oophorectomy, which leads to an immediate drop in both estrogen and androgen levels, had a significant increased risk of developing cognitive impairment or dementia; but women received hormone replacement therapy from the time of surgery did not have any increased risk [48]. Furthermore, loss of neuroprotective progesterone may attribute to the development of AD in post-menopausal women [49,50]. Estrogen may exert neuroprotective influence through mediating apolipoprotein E (apoE) encoded by APOE gene. ApoE protein encoded by wild APOE genotype promotes neurite outgrowth and neural regeneration [51,52]. Estrogen increases the level of apoE protein, and as a result, provides neuroprotection. In this trial, the female cohort had a mean age of 73.8 (51-85, SD = 8.4). Majority of the female subjects have undergone menopause for decades, which brought the sex hormones to a similar level with male subjects, and this may have negated the neuroprotective feature of estrogen and progesterone.

The APOE4 allele of APOE gene is the strongest genetic factor for sporadic AD, and it confers greater risk for female carriers to develop MCI or AD than non-carriers or male carriers [27,28], especially in impairment of verbal memory and learning ability [53]. Female carriers of APOE4 allele have greater atrophy in the hippocampus and cortex, less neural connectivity, and a higher level of neurofibrillary tangles - characteristic findings in tau pathology dementias - which is likely modulated through tau pathology [54-57]. In animal research, tNIR treatment-induced reduction of hyperphosphorylated tau protein, which gives rise to neurofibrillary tangles [58,59]. Although no direct evidence pointed towards modulation of APOE4 allele expression by PBM treatment, researches have demonstrated that gene expression can be modulated by PBM on a cellular level [60,61]. In this trial, subjects did not go through a genetic diagnostic sequencing, therefore, it is unclear to us about the patient genetic profile. Yet this warrants further study both in bench research and clinical trials to better understand PBM effects on APOE4 expression.

Studies examining gender differences on the incidence of dementia have often yielded contradicting results. In the Swedish Twin Registry study of dementia, the incidence of any dementia is similar in both sex until after age 80, where dementia rate is higher in women than men [46]. This has been attributed to the greater longevity in women. Others have demonstrated sex-specific difference in different types of dementia. Alzheimer’s Dementia affects more women than men at any given age [42]; Frontotemporal Dementia is more prominent in male than female [62]; and that Parkinsons’s disease is more prevalent in men than in women [63]. When it comes to mortality, however, women have presented lower mortality rate regardless of cognitive levels compared to men. Compared to women, men have higher mortality rate with AD, VaD, and mixed dementia, except FTD and LBD [64].

Apart from dementia incidence between different sexes, the neurological presentations of dementia have also been investigated. Semantic memory impairment is one of the earliest manifestations in AD, it has been observed in up to 50% of MCI as well [65]. In healthy individuals, sex-by-domain interactions in semantic subcategories have been documented. Men tend to perform better in some nonliving subcategories, and while women outperform in some living subcategories [66,67]. AD has category-specific effect in naming tasks on subjects. Javier et. al. examined the impact of sex on category fluency on AD subjects and found that female AD subjects demonstrated smaller fluency in almost every subcategory after controlling for disease severity and level of education [68]. This aligns with the more pronounced impact of APOE4 on female AD subjects.

Although without statistical significance, sex appears to correlate with a specific aspect of neurocognitive performance. Female performed nominally better in both CDT and CCT; while male performed nominally better in both LMT-I and LMT-II. This could be attributed to the cognitive processing characteristics of each test. In the Clock Copying and Drawing Tests, subject resorts to a more comprehensive sequence of cognitive domains involving memory and concentration, verbal understanding, abstract thinking, planning, visuospatial and constructive skills [69]. In comparison, Logical Memory Test involves less planning and visuoconstructive skills.

During the trial, mood elevation had been observed either through direct assessor-patient interaction or reports from dedicated caregivers. This has been reported more often in people receiving active PBM treatment. Contrary to traditional understanding, depression acts as a marker of MCI rather than a risk factor for response to cognitive decline, which has been attributed to the common underlying pathophysiological process [70-72]. Fuhrer et al. demonstrated that in patients with low depressive symptomology, effect of depression is only associated with MCI in women, this could be explained by the social sex stereotype that men are less willing to admit to a depressive mood; while interpersonal challenge associated with depression found to be significant in men with MCI only [73]. The somatic effect of depression dimension was significantly associated in both men and women. Due to dissimilar study designs, populations, education and baseline cognition status, researches examining the association between depression and dementia in different sexes have produced contradictory results as well [70,73,74]. Nonetheless, the assessment of specific depressive symptoms could facilitate identifying high-risk AD dementia profiles likely to benefit from interventions; similarly, changes in depressive symptoms could reflect early response to dementia intervention or treatment and should therefore be monitored accordingly.

The importance of preventive care has long been well recognized in modern medicine and is more so the case with regard to dementia prevention and treatment. Before the diagnosis of dementia, people initially started experiencing cognitive deficit, either memory loss, mood change, or language difficulties. Not meeting the full criteria of dementia, they are diagnosed with MCI. At this stage, the underlying pathologies are not fully developed and have limited scope, specificity and severity of functional impairment. Therefore, patients present with overlapping symptoms associated with a number of different pathological conditions. As the disease progresses, more extensive impairment occurs in different cognitive realms, and neuropsychologists can categorize the underlying pathology. The time from MCI to full-blown clinical dementia ranges from 3.1 to 4.1 years on average, and gender differences do exist. Norton et. al. demonstrated that it takes less time for men than women to develop full-blown clinical dementia once being diagnosed with MCI, with a median duration of 3.5 (3.1-4.0) years for men, and 3.6 (3.2-4.1) years for women [73]. This timeframe from MCI to clinical dementia is substantial in terms of timing for introducing PBM or other neurostimulation or neurocognitive training interventions. Given the different timelines from MCI to dementia in men and women, loss of estrogen-associated neuroprotection after menopause, sex-specified tNIR treatment regimen should be incorporated to maximize the beneficial effect of PBM and related interventions on dementia prevention and treatment.

Limited attention has been given to gender differences in caregivers of people struggling with dementia. Of the total 60 caregivers in this trial, 73.3% were female, and 26.7% were male. It was noteworthy that all male subjects were cared for by a female caregiver, while only 66.7% female subjects were cared for by a male caregiver. Researchers in dementia tend to use gender-neutral terms to refer to caregivers. However, dementia care differs between male and female caregivers. Men who hold to traditional gender role stereotypes would likely see caregiving a feminine role behavior, which conflicts with the traditional perception of masculinity. This conflict could make it difficult to adopt and commit to a caregiver role [75]. This view is shared in a number of Western cultures and as a result, male caregivers are likely to receive support in caregiving, usually from a female family member [76]. In contrast, women perceive the caregiver role as substantial as other responsibilities and obligations in daily living. This sense of responsibility oftentimes precludes them from seeking support, in turn causes greater emotional, physical and financial burden on female caregivers [77,78].

Limitations

The sample size is limited in this study. Clinical trials with larger number of subjects are warranted to further explore gender differences in the response of people diagnosed with dementia to tNIR stimulation. Emotional changes and gender-based caregiving were not quantified, associated findings could be anecdotal. Subjects were not stratified based on underlying neuropathology, i.e., different forms of dementia may respond quite differently to tNIR stimulation. Some of the cognitive assessments are inherent with ceiling and flooring effects, which could limit their abilities in detecting small changes and differences. Concurrent use of medications, such as donepezil, galantamine, and rivastigmine, were not captured in this trial, therefore, it was uncertain whether synergistic effect exists between tNIR and AD medications. 

## Conclusions

Despite anatomical, chemical, genetic and socio-psychological differences, both male and female dementia subjects responded quite positively to intensive, self-administered tNIR stimulation. We conclude that, regardless of underlying neuropathology, response to tNIR stimulation in dementia do not demonstrate gender-based differences. Further studies are warranted to investigate more thoroughly the response to tNIR intervention. New findings could offer guidance towards the development and refinement of tNIR treatment protocol for people suffering from dementia or dementia-related symptoms.
